# Carbon Dots and Graphene Quantum Dots in Electrochemical Biosensing

**DOI:** 10.3390/nano9040634

**Published:** 2019-04-19

**Authors:** Susana Campuzano, Paloma Yáñez-Sedeño, José M. Pingarrón

**Affiliations:** Departamento de Química Analítica, Facultad de CC. Químicas, Universidad Complutense de Madrid, E-28040 Madrid, Spain; yseo@quim.ucm.es

**Keywords:** carbon dots, graphene quantum dots, electrochemical biosensing, electrode modifiers, nanocarriers

## Abstract

Graphene quantum dots (GQDs) and carbon dots (CDs) are among the latest research frontiers in carbon-based nanomaterials. They provide interesting attributes to current electrochemical biosensing due to their intrinsic low toxicity, high solubility in many solvents, excellent electronic properties, robust chemical inertness, large specific surface area, abundant edge sites for functionalization, great biocompatibility, low cost, and versatility, as well as their ability for modification with attractive surface chemistries and other modifiers/nanomaterials. In this review article, the use of GQDs and CDs as signal tags or electrode surface modifiers to develop electrochemical biosensing strategies is critically discussed through the consideration of representative approaches reported in the last five years. The advantages and disadvantages arising from the use of GQDs and CDs in this context are outlined together with the still required work to fulfil the characteristics needed to achieve suitable electrochemical enzymatic and affinity biosensors with applications in the real world.

## 1. Introduction

The increasingly challenging demands of molecular determination have led to great research efforts to improve the analytical performance of electrochemical biosensors, enhancing their unique features for practical point-of-care (POC) analysis in terms of cost and portability and for direct analysis in complex biological matrices even for multiple target analytes. So far, this goal has been achieved mainly in connection with the use of rational surface chemistries, such as diazonium salts chemistry, and/or smart nanomaterial integration. Nanomaterials have been employed as electrode modifiers to increase the immobilized bioreceptor loadings, as electroactive labels, as nanocarriers for signaling elements (detector bioreceptors, electroactive label, and/or enzyme molecules), or a combination of these uses to achieve signal amplification. 

It is well known that nanostructuration of working electrodes with nanosized carbon modifiers provides attractive new features, such as easy physical adsorption onto electrodes through van der Waals forces; fast electron transfer kinetics due to their extremely high conductivity along particular directions; high surface area, which enhances biomolecules’ adsorption; highly selective and tunable catalysts due to their unique electronic or plasmonic structure; and tunable surface chemistry towards the direction of the assembly for particular capture probe or analyte species [1]. In addition, their excellent biological compatibility, ease of preparation, and organic functionalization make carbon-based nanomaterials excellent carriers for loading numerous signal elements, such as enzymes, oligonucleotides, antibodies, redox molecules, and inorganic nanomaterials, to greatly amplify the transduction signals of recognition events in electrochemical bioassays [2].

**Figure 1:**
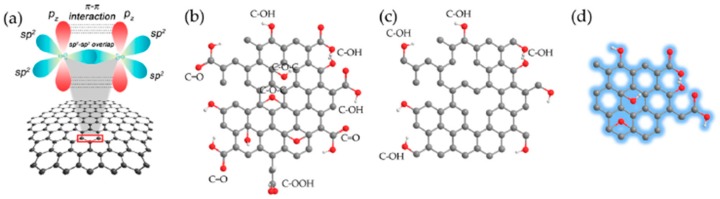
Structures of graphene-based nanomaterials, including pristine graphene (pure-arranged carbon atoms) with sp^2^-hybridized carbon atoms (**a**) and the chemically modified graphene: graphene oxide (GO) (**b**), reduced graphene oxide (rGO) (**c**), and graphene quantum dot (GQD) (**d**). Reprinted and adapted with permission from [4]. Copyright MDPI, 2017.

Among the recently developed carbon nanostructures, carbon dots (CDs) and graphene quantum dots (GQDs) are among the latest research frontiers in carbon-based nanomaterials and have attracted great interest in the development of electrochemical biosensors [3]. Figure 1 shows the major types of graphene-based materials used in engineering electrochemical biosensors. 

Due to their unique properties, carbon nanotubes (CNTs) and graphene have been widely utilized for the preparation of modified electrodes, overcoming some drawbacks of conventional carbon materials, such as a hindered electrical conductivity and mechanical strength. However, these carbon nanomaterials may suffer from problems derived from the difficult fabrication and high costs for commercial production [5]. GQDs are a kind of zero dimensional (0D) nanostructure from the carbon family with characteristics derived from both graphene and CDs. By converting 2D graphene sheets into 0D GQDs, these nanomaterials exhibit new properties due to quantum confinement and edge effects, which are similar to CDs. Nevertheless, unlike CDs, GQDs possess a graphene structure inside the dots, regardless of the dot size, in the form of various layers that are all less than 10 nm thick and 100 nm in lateral size, which endows them with some of the unusual properties of graphene [6]. Furthermore, GQDs are recognized as good electron transporters, expanding the contact area with the analyte, which increases the electrochemical active surface area that is able to interact with some electroactive species promoting direct electron transfer (DET) from enzymes and proteins. Since the geometric surface area is a very important parameter in electrochemistry, modification of different substrates with GQDs can increase the rate of electrochemical reaction [6]. GQDs usually contain functional groups, such as carboxyl, hydroxyl, carbonyl, or epoxide, at their edges and basal plane that can act as reaction sites [7]. Regarding CDs, they are quasi-spherical nanoparticles less than 10 nm in diameter that exhibit good solubility in various solvents, desirable biocompatibility, and nontoxicity. 

Although both types of carbon quantum dots possess interesting features [8], GQDs, whose outstanding properties are discussed more in detail in the following section, have been used much more than CDs in electrochemical biosensing. GQDs are an advantageous option in the design of nanomaterial-based biosensors because of their high surface area with larger length-to-diameter ratio (the ratio of length to thickness), low intrinsic toxicity, chemical inertness, mechanical stiffness, excellent solubility, high stability, photoluminescence, easier grafting of their surface with receptors, and greater electrical and thermal conductivities compared with conventional semiconductor quantum dots, due to the π–π bonds below and above the atomic plane [9,10,11,12].

Moreover, although this aspect is out of the scope of this review, other authors have recently reported the exploitation of the exceptional properties of GQDs as electrode modifiers, alone or in connection with chitosan, molecular imprinting technology, carbon based hollow-structured nanospheres (HNSs), nanochannels of vertically-ordered mesoporous silica, peptide nanofibers (PNFs), and graphene oxide (GO) nanosheet for non-enzymatic electrochemical sensing of different types of analytes [13], including doxorubicin [14], etoposide [15], L-cysteine [16], phenols [17,18,19] Hg^2+^, Cu^2+^, Cd^2+^ [20], Cr^6+^ [21], dopamine [19,22,23], H_2_O_2_ [24,25,26,27,28], etc., in a wide range of samples (fruit juices, honey, seafood, soil leaching solution, human serum and plasma, living cells). GQDs have also been used as electrode modifiers in enzymatic and affinity biosensors (immunosensors, nucleic acid sensors, and aptasensors) and much less often as nanocarriers in electrochemical affinity sensors. 

This review discusses relevant advances and strategies reported over the last five years in the use of GQDs or CDs for the design of electrochemical biosensors, with special attention to representative electrochemical affinity biosensing platforms that take advantage of the excellent properties offered by these nanomaterials either as electrode modifiers or signal tags. The most remarkable advantages offered by these carbon nanomaterials in the development of electrochemical (bio)sensors compared to the widely used CNTs arise from their many surface functional groups, such as carboxyl, which impart better solubility in many solvents and aqueous media and facilitate their easy functionalization with organic, polymeric, inorganic, or biological species without further modification and/or activation. The final section discusses the main challenges to be met to take full advantage of these properties in electrochemical biosensing as well as the possible future trends in this field on the basis of the great advances that have occurred in recent years.

## 2. CDs 

CDs are defined as nanoparticles, mainly composed of carbon, with a size below 10 nm. They have aroused intense interest because of their photoluminescence [29] and photophysical properties: For example, high photostability, which resemble in some respects those found in semiconductor quantum dots (QDs), and photocatalytic applications [30]. In addition, CDs can be produced easily from a wide range of raw materials and are particularly attractive due to their robust chemical inertness and the many surface carboxylic acid moieties, which provoke excellent water solubility and facilitate their subsequent functionalization with organic, polymeric, inorganic, or biological species. CDs have been proposed as promising substitutes for traditional QDs because they do not require tedious and costly preparation steps and possess high biocompatibility due to the absence of toxic metal ions. Regarding CDs’ preparation, a variety of methods, including acidic oxidation, microwave, ultrasonic, electrochemical oxidation, hydrothermal, supported synthesis, arc discharge, and laser ablation, have been reported. However, unlike other carbon based nanomaterials and despite the interesting features, CDs have not been widely explored as electrode modifiers for the development of electrochemical biosensors.

The few applications of CDs in electrochemical biosensing described to date are mainly focused on the electrocatalytic properties of these nanoparticles towards O_2_ [31] and H_2_O_2_ reduction, exploited for the biosensing of glucose [32,33] or H_2_O_2_ [34] and the sensing of dopamine [35], 2,4,6-trinitrotoluene [36], patulin [37], and glucose [38]. In the particular case of electrochemical affinity biosensors, only one example involving the use of CDs as electrode modifiers in the preparation of a DNA sensor to detect single gene mutations has been reported so far [1]. It is worth mentioning that, to our knowledge, the use of CDs as labels in electrochemical biosensors has not been described yet. 

## 3. GQDs 

The zero bandgap graphene prevents it from having an exciton and emitting light on excitation. Therefore, by cutting down the graphene to a small size, non-zero band gap materials, like GQDs, are formed and can behave like semiconductors. GQDs are graphene sheets of less than 100 nm, which have a larger specific surface area, more surface active sites, and more accessible edges than graphene [39]. Due to the quantum confinement and edge effect, GQDs have high speed electron transport and excellent conductivity, which make them a relevant transducer material to enhance or control the heterogeneous electron transfer for the design of (bio)sensors besides their optical properties. The electronic and optical properties of GQDs depend on the shape, height, and edge states of GQDs.

These nanoparticles (NPs) can be obtained as single-layer, double-layer, and multi-layer materials, and they possess interesting properties to be used in the development of electrochemical biosensors, such as robust chemical inertness, excellent photostability, high biocompatibility, and low toxicity [7]. 

GQDs also contain at their edges carbonyl, hydroxyl, and amino functional groups, which impart excellent solubility in aqueous media and have an ability to functionalize with organic, inorganic, or biological moieties, which is essential for the development of electrochemical biosensors [9,40,41,42,43]. Therefore, in the last years, GQDs have been employed as relevant nanomaterials to replace inorganic semi-conductive QDs as label tags due to their low toxicity and easy functionalization. 

GQDs are recognized as good electron transporters and acceptors and show peroxidase-like properties [44,45,46,47,48], exhibiting electrochemical catalytic properties toward H_2_O_2_ decomposition and allowing its sensitive detection [25,27,45], even in living cells after covalent assembly on Au [6,24]. Moreover, GQDs may in the future replace graphene, GO, or rGO as an immobilized template given that GQDs can overcome some of the disadvantages associated to the non-conducting nature of graphene oxide, and the lack of functional groups for biomolecule attachment in pure graphene [7,9,40]. It is important to remark that functionalization requires the creation of –COOH or –NH_2_ groups on the graphene forms to support biomolecules’ attachment, but it may also affect their electrochemical properties [40]. Moreover, due to oxygenated functional groups and a rich peripheral carboxyl cornerstone with a relatively complete aromatic planar structure, GQDs can oxidize 3,3´,5,5´-tetramethylbenzidine, hydroquinone (HQ), and other redox mediators, thus showing peroxidase-like activity [42,45].

Similar to graphene, GQDs can be grafted with various functional groups through π-π conjugated network [40]. Indeed, the structural similarity of GQDs and CNTs makes an interaction between both nanomaterials possible through strong π-π interactions, resulting in a hybrid nanomaterial that merges the fascinating properties of the individual components [47,48]. Moreover, doping heteroatoms into the π-conjugated system of GQDs was shown to be a very effective strategy to improve GQDs’ intrinsic properties, such as chemical stability, electrical conductivity, and electrocatalytic activity. For example, in nitrogen-doped graphene quantum dots (N-GQDs), the chemically bonded N atoms alter the electronic characteristics and increase the number of anchoring sites for the adsorption of metal ions, such as bimetallic nanoparticles, to provide superior auxiliary catalytic activity for oxygen and H_2_O_2_ reduction reactions [23,49]. 

Unfortunately, there is not yet a great variety of commercially available GQDs, and their synthesis involves long and laborious procedures with (sometimes) a lack of reproducibility. In general, the synthesis of QDs with an exact size is not easily reproducible, and there is a possibility of agglomeration and the formation of large-sized QDs [42].

At present, there are mainly two developed routes for GQDs’ preparation: ‘Top-down’ and ‘bottom-up’ methods. Top-down methods involve a nanosize carving of carbon materials through chemical or physical routes (ionic liquid assisted grinding, hydrothermal, chemical ablation, photofenton oxidation, oxygen plasma treatment, electrochemical oxidation, etc.). On the contrary, bottom-up methods are based on the carbonization of an organic precursor by means of a thermal treatment (microwave, solvothermal treatment, thermal pyrolysis, etc.). There are obvious advantages of bottom-up methods over top-down methods, because they allow precise control of the composition and physical properties of GQDs by careful selection of the precursors and carbonization conditions [23,39,50].

The following sections highlight representative strategies involving mostly GQDs, but also CDs, in electrochemical biosensing related to the preparation of both enzymatic and affinity biosensors. 

## 4. GQDs and CDs in Electrochemical Biosensing 

The excellent properties provided by GQDs and CDs for the immobilization of bioreceptors and for the electrocatalysis of many relevant compounds (H_2_O_2_, O_2_, dopamine, etc.) mean that such nanomaterials are more frequently used in electrochemical biosensing mainly as electrode modifiers, but also, and increasingly, in regards to GQDs, as advanced labels [13]. 

### 4.1. Electrochemical Enzymatic Biosensing Involving GQDs 

Taking advantage of the large surface-to-volume ratio, excellent biocompatibility, abundant hydrophilic edges, and a partially hydrophobic plane that favors protein adsorption [51], GQDs have been used as electrode modifiers in the preparation of electrochemical enzyme biosensors. Moreover, electrode modification with GQDs preserves the functionality of the immobilized enzyme, enhances the electrochemical signal, and the nanoscale dimension of GQDs permits the wiring of enzyme redox-centers and, therefore, DET, especially of oxidoreductases, like laccase [12].

Muthurasu et al. [52] reported a horseradish peroxidase (HRP) biosensor involving covalent immobilization of the enzyme onto a glassy carbon electrode (GCE) modified with GQDs through amidic bonds between carboxylic groups of GQDs and amine groups of the enzyme. The amperometric monitoring of H_2_O_2_ provided a limit of detection (LOD) of ~530 nM. Direct electron transfer (DET) of hemoglobin (Hb) immobilized on a nanocomposite of GQDs-chitosan (GQDs-Chit) was employed to construct a biosensor for the cyclic voltammetry (CV) determination of H_2_O_2_ [53]. This Hb-biosensor provided a linear range between 1.5 and 195 *μ*M, an LOD of 0.68 *μ*M for H_2_O_2_, and was successfully applied to the determination of H_2_O_2_ in urine samples.

An electrochemical laccase biosensor for the determination of the polyphenol index in red wine was proposed by Vasilescu et al. [54] by bringing together for the first time the use of molybdenum disulphide (MoS_2_) nanoflakes and GQDs as electrode modifiers of a screen-printed carbon electrode (SPCE). MoS_2_ is one of the most studied 2D layered materials after graphene and exfoliated MoS_2_ flakes have demonstrated good ability for sensing. The presence of the bandgap for MoS_2_ (of 1.8 eV for an MoS_2_ monolayer) in contrast to the zero-gap semiconductor graphene leads to electrocatalytic activity. The resulting enzyme biosensor (SPCE-MoS_2_-GQDs) exhibited improved electrocatalytic activity and conductivity compared to the individual counterparts (SPCE-MoS_2_ and SPCE-GQDs) due to synergistic interaction between the GQD and MoS_2_ sheets. This laccase biosensor relied on the electrostatic interactions of the enzyme at the surface of the MoS_2_/GQDs/SPCE and showed an attractive performance for the chronoamperometric determination of caffeic acid, chlorogenic acid, and epicatechin, achieving LODs of 0.32, 0.19, and 2.4 μM, respectively. 

DET glucose biosensors were prepared by physical adsorption of glucose oxidase (GOx) on GQDs casted at the carbon ceramic electrode (CCE) [55] and GCE [51]. The resulting biosensors showed an excellent performance for the amperometric determination of glucose with LODs in the low μM level, 1.73 μM and 1.35 μM, respectively. Interestingly, the biosensors prepared using GQDs/GCE behaved better than their counterparts prepared using GO and rGO nanosheets, yielding LODs of 4.82 and 4.16 μM, respectively. 

Baluta et al. [12] have recently reported an electrochemical biosensor for epinephrine (EP) through the immobilization of laccase by cross-linking with glutaraldehyde on a GQDs/GCE. By monitoring the oxidation of catecholamine by CV, the DET-based laccase biosensor exhibited a broad linear range (1–120 μM), an LOD of 83 nM, and successful applicability for EP determination in pharmacological samples.

### 4.2. Electrochemical Affinity Biosensing Involving GQDs or CDs

The interesting characteristics of GQDs both as electrode materials and labels have been exploited in the field of electrochemical affinity sensors, more frequently dealing with immunosensors, but also in nucleic acid sensors for the determination of DNAs and microRNAs, and still rarely in aptasensors [6,11]. It is worth remarking that so far, only one electrochemical DNA sensor has been reported using CDs as an electrode modifier [1]. 

#### 4.2.1. GQDs in the Preparation of Electrochemical Immunosensors 

The high surface area and abundance of functional groups in GQDs as well as their easy functionalization and attractive electrochemical properties, similar to graphene, have led to the use of GQDs as surface electrode modifiers and as carrier tags for signal amplification in the development of electrochemical immunosensors.

The main features of the electrochemical immunosensors reported so far involving GQDs are summarized in Table 1. They mostly imply the use of this nanomaterial as an electrode modifier, although very recently, GQDs have also been employed as nanocarriers. Regarding the role of an electrode modifier, they have been used as single, hybrid, or nanotriplex materials to immobilize the capture antibody (CAb). Although most of the reported methods are label-free, the excellent electrocatalytic activity of GQDs towards H_2_O_2_ reduction has also been exploited using amperometric detection. 

Valipour and Roushani [56] reported the use of a nanocomposite of silver nanoparticles (AgNPs) and thiolated graphene quantum dots (GQD-SH) as a GCE modifier to develop a label-free immunosensing platform for hepatitis C virus core antigen (HCV). AgNPs were immobilized on SH groups of GQDs via Ag-S bonding formation and CAb loading on the AgNPs through its –NH_2_. The immunorecognition reaction was monitored by measuring the decrease in the oxidation signal of riboflavin using differential pulse voltammetry (DPV). The immunosensor showed a wide linear range (0.05 pg mL^−1^ to 60 ng mL^−1^), an LOD of 3 fg mL^−1^, and was applied to the analysis of spiked human serum.

**Figure 2:**
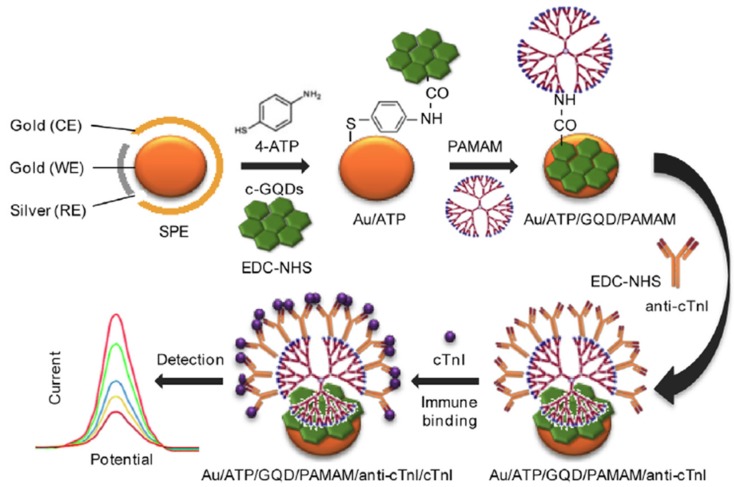
Electrochemical immunosensor constructed for the determination of cTnI involving the use of an Au/GQD/PAMAM nanohybrid electrode. Reprinted with permission from [10]. Copyright Elsevier, 2017.

Bhatnagar et al. [10] developed an ultrasensitive electrochemical immunosensor for cardiac troponin I (cTnI) using nanohybrids of GQDs and polyamidoamine (PAMAM) to provide an ultra-high surface area for antibody immobilization as electrode modifiers. In this method, GQDs were covalently coupled through 1-Ethyl-3-(3-dimethylaminopropyl)carbodiimide/ N-Hydroxysuccinimide (EDC/NHS) chemistry on an amino functionalized Au screen-printed electrode. 4-aminothiophenol (4-APT) and the poly(amidoamine) (PAMAM) dendrimer were successively attached to GQDs through EDC coupling to provide an ultra-high surface area for the immobilization of the capture antibody (Figure 2). The recognition of the target protein was monitored by the decrease in the Fe(CN)_6_^3-^ oxidation peak measured by DPV and CV, achieving LODs of 20 and 25 fg mL^−1^, respectively. 

Other label-free electrochemical immunosensors have been reported for the determination of myoglobin (cMyo) [40], parathion [9], and the receptor tyrosine kinase AXL, a relevant biomarker in cancer, inflammatory processes, and heart failure [43], at screen-printed carbon electrodes (SPCEs) modified with GQDs. While in the former immunosensor the CAb was covalently immobilized using EDC/NHS chemistry onto the GQDS surface confined –COOH groups [40], the other two immunosensors involved the grafting of GQDs with 2-aminobenzyl amine (2-ABA) and CAb immobilization through stabilization of the Schiff bases between the aldehyde groups, generated on the antibody by periodate-mediated oxidation of their carbohydrate moieties, and the NH_2_-functionalized GQDs [9,43]. The immunorecognition of the target analyte was followed by electrochemical impedance spectroscopy (EIS) [9] or DPV [43] in the presence of [Fe(CN)_6_]^3-/4-^, or by monitoring the reduction peak of the heme group (Fe^3+^) of the captured cMyo using DPV [40]. The three immunosensors exhibited wide linear ranges and low LODs (see Table 1). In addition, the immunosensor for parathion could be re-used during five regeneration cycles and the immunosensors for AXL and cMyo were successfully applied to the determination of the cardiac biomarkers in spiked human serum [40] and in serum from heart failure (HF) patients [43]. 

**Figure 3:**
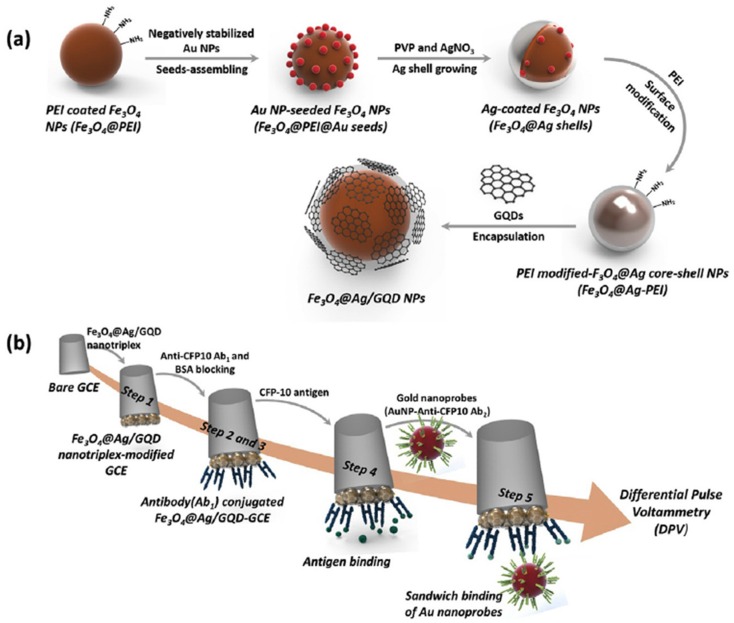
Synthesis of Fe_3_O_4_@Ag/GQD NP nanotriplex probe (**a**) and outline of inmmunosensor preparation (**b**): drop casting of the nanotriplex probe onto the GCE (Step 1), incubation with CAb (Step 2), blocking with BSA (Step 3), incubation with the target antigen (Step 4), and incubation with the AbD-AuNPs (Step 5). Reprinted with permission from [57]. Copyright Elsevier, 2018.

The use of GQDs nanotriplex materials as GCE modifiers has also been exploited in the development of attractive direct or sandwich-based electrochemical immunosensing platforms. A label-free electrochemical immunosensor was reported for the detection of carcino-embryonic antigen (CEA) using nanocomposites of N-GQDs, PtPd bimetallic nanoparticles, and Au nanoparticles (PtPd/N-GQDs@Au), which showed excellent electrocatalytic activity towards H_2_O_2_ reduction [49]. By using amperometry in the presence of H_2_O_2_, the immunosensor provided a wide dynamic range (5 fg mL^−1^–50 ng mL^−1^), a low LOD (2 fg mL^−1^), and could be used for the analysis of spiked human serum samples. Tufa et al. [57] proposed an electrochemical sandwich immunosensor for the detection of *Mycobacterium tuberculosis* antigen (culture filtrate protein, CFP-10) using a nanotriplex consisting of a GQD-coated Fe_3_O_4_@Ag core-shell nanostructure (Fe_3_O_4_@Ag/GQD) as a the GCE modifier and AbD-AuNPs as labels for signal amplification (Figure 3). The sensing platform using the nanotriplex material showed a noticeable synergetic electrochemical performance due to the different roles of the three nanomaterials: Fe_3_O_4_ increased the surface-to-volume ratio; Ag enhanced electrical conductivity; and GQDs allowed larger CAb loadings onto the electrode. The sandwich immunocomplexes were electrochemically pre-oxidized in the presence of 0.1 M HCl and the Au^3+^ generated was then reduced to Au^0^ using DPV. The immunosensor exhibited a wide linear range (0.005–500 mg mL^−1^) and an LOD of 0.33 ng mL^−1^, and was successfully applied to the analysis of spiked human urine samples. 

**Figure 4:**
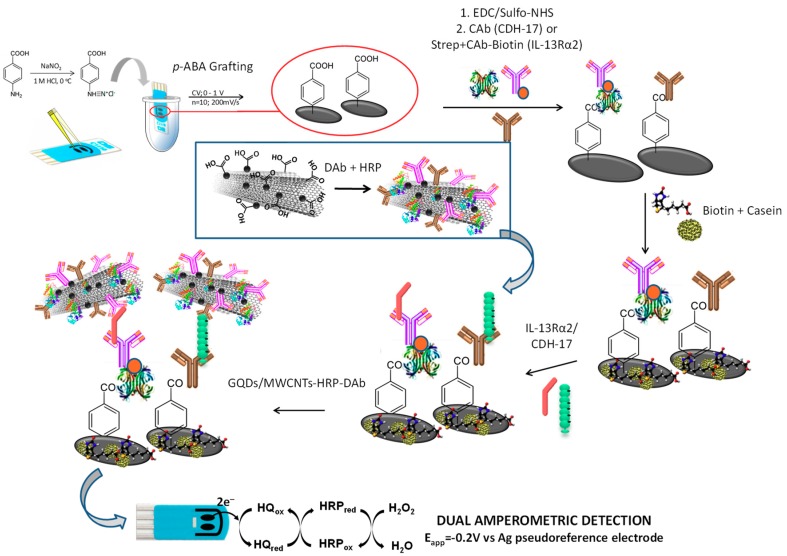
Steps involved in the preparation and functioning of the dual immunosensing platform developed for the amperometric determination of the target biomarkers, IL-13Rα2 and CDH-17, involving the use of MWCNTs/GQDs-HRP-DAb nanocarriers. Figure drawn by ourselves based on [48].

Very recently, Pingarrón´s group has proposed the use of hybrid nanomaterials composed of muti-walled carbon nanotubes (MWCNTs) and GQDs as nanocarriers of detector antibody (Dab) and horseradish peroxidase (HRP) molecules in sandwich immunosensors prepared for the single [47] or dual [48] determination of the emerging metastasis cancer biomarkers, IL-13 soluble receptor Rα2 (IL-13sRα2) and cadherin-17 (CDH-17). The integrated immunosensing scaffolds involved immobilization of biotinylated or non-biotinylated CAbs onto SPCEs grafted with *p*-aminobezoic acid (*p*-ABA), activated using EDC/Sulfo-NHS chemistry and modified with Streptavidin (in case of biotinylated CAb). Once the sandwich immunoassays were implemented with the corresponding MWCNTs/GQDs-HRP-DAb nanocarriers, an amperometric transduction was accomplished in the presence of the H_2_O_2_/HQ system at single or dual SPCEs (SPCE and SPdCE, respectively) (Figure 4). The prepared scaffolds allowed the implementation of the first integrated immunosensors reported so far for the determination of these emerging biomarkers. In addition, it was the first time that MWCNTs/GQDs were used as nanocarriers in connection with electrochemical immunosensing, confirming the intrinsic peroxidase-like catalytic activity of GQDs. The developed immunosensors were applied to the determination of the target biomarkers directly in small amounts (0.5 μg) of raw cellular lysates and extracts of paraffin-embedded tissues from patients diagnosed with colorectal cancer (CRC) at different stages and even in whole cells. 

#### 4.2.2. Electrochemical Nucleic Acid Biosensors and Aptasensors Involving GQDs or CDs

GQDs have been much less used for the development of DNA sensors than for immunosensors. Nevertheless, they have also demonstrated interesting features for the electrochemical biosensing of nucleic acids (both of DNA or RNA nature) when used as artificial enzymatic tracers, nanocarriers, and electrode modifiers to immobilize specific capture probes (Cps) and to enhance the electroactivity of redox molecules. The main characteristics of the reported nucleic biosensors are summarized in Table 1. 

**Figure 5:**
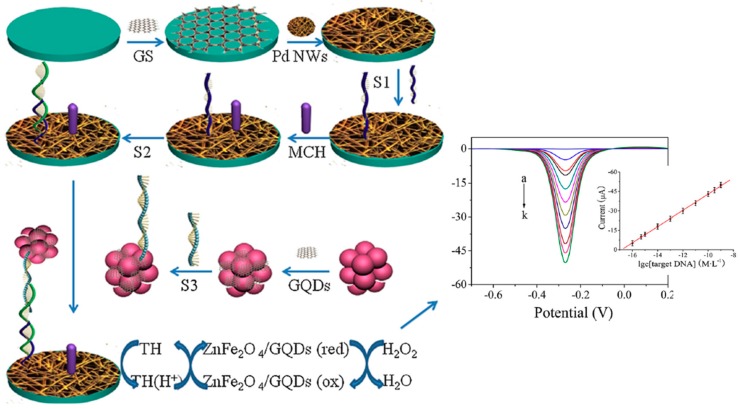
Schematic illustration of the electrochemical sandwich hybridization DNA sensor involving the use of ZnFe_2_O_4_/GQDs as a mimicking trace label and a nanocarrier of Dp (S3); real DPV responses of the DNA biosensor in the presence of different target DNA (S2) concentrations (0, 10^−16^, 5 × 10^−15^, 10^−15^, 10^−14^, 10^−13^, 10^−12^, 10^−11^, 10^−10^, 5 × 10^−10^, and 10^−9^ M, a→k) and resulting calibration curve. Reproduced and adapted with permission from [45]. Copyright Elsevier, 2014.

Liu et al. [45] reported an electrochemical sandwich hybridization DNA sensor using magnetic ZnFe_2_O_4_/GQDs nanohybrids both as a mimic peroxidase-like enzymatic label and as a nanocarrier of a detector DNA probe (S3). A specific capture DNA probe (S1) was immobilized onto a GCE modified with aminated graphene and Pd nanowires and the captured target DNA (S2, not related to any particular analyte) was sandwiched with the Dp/ZnFe_2_O_4_/GQDs. The ZnFe_2_O_4_/GQDs nanohybrid exhibited a highly-efficient peroxidase-like catalytic activity, producing large DPV reduction peaks in the presence of thionine (TH) and H_2_O_2_ (Figure 5). The DNA sensor showed a wide linear range (10^−16^–5 × 10^−9^ M) and low LOD (6.2 × 10^−17^ M) as well as a good performance in the analysis of spiked human serum samples. 

A specific and sensitive method was developed for the determination of the microRNA miRNA-155, of relevance in myocarditis, by integrating the HRP assisted catalytic reaction with a simple electrochemical RNA biosensor [41]. The electrochemical biosensor for miRNA determination was constructed by using a double stranded DNA structure formed by sandwiching the target miRNA with a thiolated Cp assembled on a gold electrode surface, and an aminated Dp (NH_2_-Dp). After formation of the sandwich DNA, the GQDs, assembled through their activated carboxyl groups on the NH_2_-Dp, were used as a scaffold for HRP immobilization through non-covalent assembly. The resulting biosensor effectively catalyzed the H_2_O_2_-mediated oxidation of 3,3′,5,5′-tetramethylbenzidine (TMB), and the corresponding chronoamperometric measurements allowed sensitive detection of the target miRNA (LOD of 0.14 fM) with a linear range from 1 fM to 100 pM, as well as its reliable determination in spiked human serum samples. 

A simple label-free electrochemical platform has been designed for the detection of hepatitis B virus (HBV) DNA using GQDs as modifiers of a GCE [39]. The method used a DNA probe complementary to the HBV-DNA, which in the absence of the target DNA strongly bound to the surface of the GQDs and produced a decrease in the DPV K_3_[Fe(CN)_6_] oxidation signal. Conversely, the presence of the target HBV-DNA provoked the probe DNA to hybridize with it instead of the GQDs, thus providing increased voltammetric responses with the target DNA concentration. The sensor achieved an LOD of 1 nM and a linear detection range from 10 to 500 nM. 

**Figure 6:**
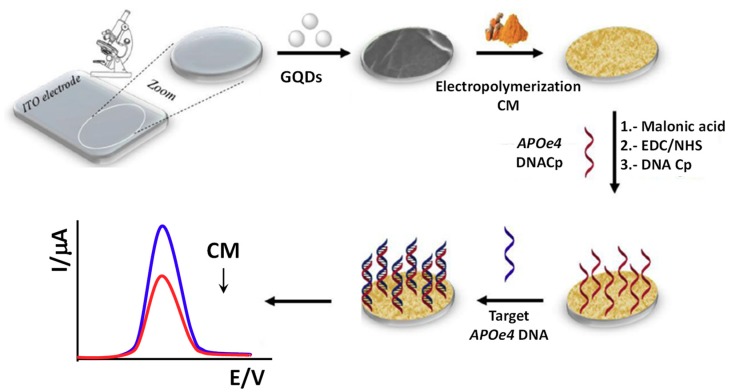
Schematic representation of the electrochemical DNA biosensor developed to sense Alzheimer’s and artery coronary diseases using CM/GQDs as surface modifier of the ITO electrode. Reproduced and adapted with permission from [58]. Copyright Elsevier, 2018.

Mars et al. [58] have proposed recently a dual fluorescence and electrochemical DNA sensor using an indium-tin oxide (ITO) electrode modified with curcumin, (CM)/GQDs, to sense Alzheimer’s and artery coronary diseases by targeting *APOe4* DNA. The CM molecule, with dual fluorescence and electrochemical properties, was electropolymerized on the GQDs-ITO surface. EDC/NHS chemistry was used to covalently immobilize an amino-substituted DNA probe via a malonic acid spacer (Figure 6). The wrinkled surface of the GQDs, rich in active carboxylic sites, allowed hydrogen bonds with CM molecules, enhancing significantly the CM electroactivity. The decrease in the CM DPV oxidation peak upon hybridization was used as the analytical readout, providing an LOD for the target DNA of 0.48 pg mL^−1^ and allowing the determination in 100-fold diluted human blood plasma pre-treated with ammonium sulfate to precipitate fibrinogen. 

**Figure 7:**
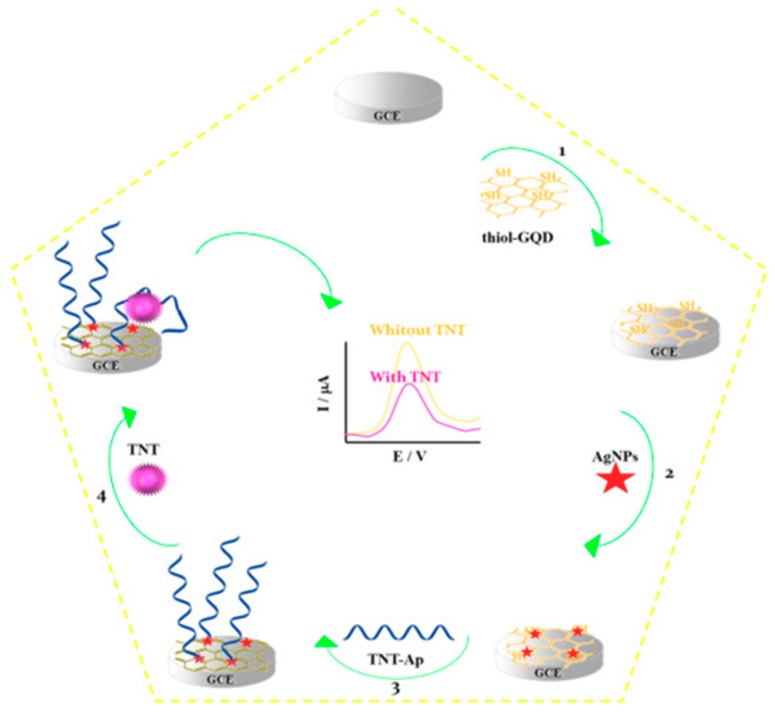
Label-free aptasensor developed for the determination of TNT by immobilizing the NH_2_-Ap onto an AgNPs/thiol-GQD/GCE and performing the DPV detection in the presence of RU. Reprinted with permission from [59]. Copyright Elsevier, 2017.

Only one electrochemical aptasensor has been reported to date involving the use of GQDs. Shahdost-fard and Roushani [59] developed a label-free aptasensor for the detection of 2,4,6-Trinitrotoluen (TNT) by immobilizing an amino-modified aptamer (NH_2_-Ap) onto a GCE modified with an AgNPs/thiol-GQD nanocomposite. It is important to note that the same group used this nanocomposite to develop immunosensing platforms [56]. AgNPs were employed as a linker between thiol-GQDs and the NH_2_-Ap and also to increase the aptamer loading on the GCE. The decrease in the DPV oxidation signal of Rutin (RU), used as a redox probe, after recognition of TNT was the analytical readout (Figure 7). The aptasensor achieved an LOD of 0.33 fM and was employed to analyze spiked soil and water samples.

García-Mendiola et al. [1] have reported the only affinity biosensor described to date involving the use of CDs. The developed electrochemical DNA sensor was designed to detect gene mutation and used screen-printed gold electrodes (Au-SPE) modified with CDs by drop-casting. DNA probes complementary to characteristic fragments of the pathogen, *Helicobacter pylori*, or the cystic fibrosis transmembrane regulator (*CFTR*) gene were adsorbed on the as prepared CDs-Au-SPE. The hybridization event with a synthetic fully complementary sequence or a PCR amplicon of exon 11 of *CFTR* containing a sequence complementary to the capture probe (of 25- and 373-bases, respectively) was monitored by DPV using safranine as the redox indicator, which selectively binds to dsDNA. The DNA sensor exhibited an LOD of 0.16 nM for the synthetic target DNA and feasibility to detect a single nucleotide polymorphism (F508del mutation in the *CFTR* gene) in human DNA extracted from blood cells.

## 5. Conclusions, Main Challenges to Solve, and Future Perspectives 

The increasing demand for sensitive and selective single or multiplexed determination of relevant analytes at different molecular levels in matrices of a diverse nature and complexity using simple and rapid protocols has led to the design and development of electrochemical biosensing platforms with improved performance. Literature reported in the last five years show that electrochemical bioassays involving GQDs and CDs provide an elegant way to fulfil these increasingly ambitious demands. These particular carbon nanomaterials have been used in electrochemical biosensing either as signal tags replacing or carrying enzymatic systems or as modifiers of the working electrode surface, always seeking signal amplification. As discussed in previous sections, the use of GQDs and CDs allows the development of sensitive, selective, and biocompatible biosensors for the detection of relevant analytes in biological matrices

GQDs and CDs have been used primarily as surface modifiers in electrochemical biosensing (enzymatic and affinity) and have been demonstrated to be good candidates to replace graphene and GO due to a set of merits, including chemically stability, excellent dispersity and solubility in aqueous and organic solvents, and efficient electrocatalytic performance (both as electron acceptors or donors) towards relevant analytes, such as H_2_O_2_ and O_2_, demonstrating their potential as artificial enzyme mimics with better stability (over broader pH and temperature ranges) than natural enzymes. 

Due to their large surface area, QGDs and CDs are very efficient for the immobilization of increased loadings of proteins, enzymes (exhibiting DET), and antibodies by simple adsorption, covalent immobilization, Schiff reaction, etc., as well as of oligonucleotides, even unmodified and maintaining their hybridization capability. In addition, their large number of functional groups allows homogeneous decoration with biomolecules and a more efficient control on the resulting biosensors’ precision and accuracy. 

It is also important to highlight the great versatility offered by these nanomaterials to be prepared, modified with different surface chemistries (such as the diazonium salts chemistry) or other organic and inorganic modifiers, and used as single or hybrid nanomaterials. 

To date, GQDs have been used, alone or in combination with MoS_2_ nanoflakes, as electrode modifiers in enzyme biosensing to increase enzyme loading, preserve enzyme functionality, and achieve DET of the enzyme immobilized by adsorption or covalent attachments. 

GQDs have also been used as modifiers of the electrode surface and as carrier tags for signal amplification in the development of electrochemical affinity biosensors. In these strategies, GQDs (or N-GQDs) have been used alone without modification or modified by grafting with diazonium salts, or as hybrid nanomaterials, such as AgNPs/thiol-GQDs, PAMAM/GQDs, MWCNTs/GQDs, ZnFe_2_O_4_/GQDs, and triplex PtPd/N-GQDs@Au, Fe_3_O_4_@Ag/GQD. The resulting affinity biosensors, involving direct or sandwich immunoassay formats, employ label-free strategies or take advantage of the excellent electrocatalytic properties of GQDs for the detection of certain redox species (H_2_O_2_, riboflavin, RU, safranine, CM). Original approaches have involved the detection of the analyte cofactor (Fe^3+^ group of the cMyo) or the Au^3+^ ions generated after the acid oxidation of the AuNPs immunoconjugates used as labels. The bioplatforms described in the last five years used conventional electrodes (mostly GCE, but also Au disk and ITO) or screen-printed electrodes (carbon or gold) and different electrochemical transduction techniques, including EIS, DPV, CV, and chronoamperometry. The implementation of one strategy involving CDs as electrode modifiers in nucleic acid sensors and a dual integrated immunosensing platform for the simultaneous determination of two protein biomarkers with relevance in metastasis is particularly remarkable. The biosensors developed so far using GQDs and CDs have focused on the determination of a wide variety of analytes, such as circulating protein biomarkers of interest in cancer and cardiovascular diseases, bacterial antigens, toxic environmental compounds, explosives, DNAs, and microRNAs, and have been applied mainly to their determination in clinical samples: Liquid biopsies (serum, urine, blood), cellular lysates, and paraffin-embedded tissues. 

It is also important to note the excellent LODs provided by the resulting biosensors, in the nM to μM level for enzyme biosensors, fg mL^−1^ to ng mL^−1^ for immunosensors, and aM to nM for nucleic acid sensors. Within this context, it should be noted that despite diffusional limitations of such low numbers of molecules to the sensor—currently overcome by nanostructuring the surfaces and/or reducing sample volumes—the need to reach low LODs is compulsory, especially in the affinity biosensing of biomarkers, which are endogenously found at very low concentrations, and/or to be able to work with diluted samples when significant matrix or (bio)fouling effects become significant. Indeed, despite these diffusional limitations at low concentrations, assay times (without counting the preparation and modification of nanomaterials and biosensing surfaces) vary between 5 min and 4 h and the most sensitive affinity biosensors summarized in Table 1 require between 10 and 160 min (immuno [10,49,56] and DNA [45] sensors) to perform the determination, testing times very competitive with conventional ELISA and PCR strategies. Some of the immunosensors have even demonstrated regeneration capabilities.

Although to date GQDs and CDs have been scarcely used as labels and not at all as signaling elements by themselves, but as carriers of such elements, these nanomaterials represent an interesting alternative to heavy-metal-based QDs due to their low toxicity and easy modification through their surface functionalities. However, unlike inorganic QDs, the applicability of GQDs as label tags for multiplexing strategies has been reported only in one work that has just come out. Moreover, the use of CDs as advanced tags has not been described so far. 

Despite the interesting features exhibited by these carbon nanomaterials in connection with electrochemical biosensing, there is still a long way to go in this field. One of the key difficulties for researchers aiming to develop electrochemical biosensors using GQDs and CDs is to obtain high-quality products. The existing synthesis methods generally allow their small-scale production and with a wide size distribution. Therefore, the search for synthetic methods that improve the nanoparticles’ quality and the production yield, allowing their purification in a simple way, is highly desirable.

It is also important to point out that, as it is clearly shown in the information summarized in Table 1, the preparation and modification of these nanomaterials require multi-step and long experimental protocols, implying, especially in the case of hybrid nanomaterials, preparation times between 11 and 56 h. In addition, further efforts should be devoted to evaluating their reproducibility, storage stability, and (bio)fouling properties, so far not thoroughly evaluated by researchers. These challenges must be addressed imminently to guarantee that the developed methods meet the demands of POC and routine determinations, thus making them suitable for translation from the laboratory to applications in the real world, some of which demand biosensors of continuous and calibration-free operation in undiluted complex samples. 

Moreover, there are still few reports in which electrochemical biosensors using GQDs or CDs have been applied to the analysis of real patient samples. So far, they have been applied mainly to spiked samples or to a limited number of real samples. Only one method has been reported involving multiplexed detection. 

Despite all the challenges that are faced, the versatility of use and modification and the excellent properties demonstrated by of GQD or CDs and their hybrid materials, coming from the synergetic effect of the individual components, awakens the interest to continue exploring new uses, modifications, and combinations of these carbon nanomaterials to facilitate the development of universal electrochemical biosensing strategies that can be applied for a whole category of analytes. Although the exploration of these nanomaterials in electrochemical biosensing still remains at an early stage and has been much lower than in optical biosensing, it is expected that the interesting advances shown so far will open new opportunities for further developments and promote the application of this star carbon nanomaterial in future electrochemical biosensing at different molecular levels. These opportunities should also pay attention to necessary efforts for facilitating their transition to the real world and competing well with corresponding fluorimetric sensors, particularly into practical POC applications. Moreover, the peroxidase-mimicking functions of these carbon nanomaterials reveals their potential applications beyond electrochemical biosensing.

## Figures and Tables

**Table 1:** Electrochemical affinity biosensors reported over the last five years involving the use of GQDs or CDs.

Electrode	Bioassay format	Nanomaterial(Role)	Target	Technique	Linear range	LOD	Assay time	Sample	Reference
GCE	Direct immunosensor	AgNPs/thiol-GQDs (electrode modifiers)	HCV	DPV (riboflavin)	0.05 pg mL^−1^–60 ng mL^−1^	3 fg mL^−1^	30 min + AgNPs (2 h) + Thiol-GQDs (54 h) + CAb/AgNPs/thiol-GQD/GCE (>3 h)	Spiked human serum	[56]
Au-SPE	Direct immunosensor	PAMAM/GQDs (electrode modifiers)	cTnI	CV, DPV (Fe(CN)_6_^3-^)	1.0 10^−6^–1.0 × 10^−3^	20 fg mL^−1^ (CV) 25 fg mL^−1^ (DPV)	10 min once prepared the Au/GQD/PAMAM/CAb (30 h)	Human blood serum	[10]
SPCE	Direct immunosensor	GQDs (electrode modifiers)	cMyo	DPV (Fe^3+^ group of the cMyo)	0.01–100 ng mL^−1^	0.01 ng mL^−1^	10 min once prepared the CAb/GQDs/SPCE (1 h 10 min)	Spiked human serum	[40]
SPCE	Direct immunosensor	GQDs grafted with 2-ABA (electrode modifiers)	Parathion	EIS ([Fe(CN)_6_]^3-/4-^)	0.01–10^6^ ng L^−1^	46 pg L^−1^	5–30 min once prepared the CAb/GQD/SPCE (~3.5 h)	—	[9]
SPCE	Direct immunosensor	GQDs grafted with 2-ABA (electrode modifiers)	AXL	DPV ([Fe(CN)_6_]^3-/4-^)	1.7−1000 pg mL^−1^	0.5 pg mL^−1^	90 min once prepared the CAb/GQD/SPCE (~4 h)	Human serum	[43]
GCE	Direct immunosensor	PtPd/N-GQDs@Au (electrode modifiers)	CEA	Amperometry (H_2_O_2_)	5 fg mL^−1^–50 ng mL^−1^	2 fg mL^−1^	1 h + CAb/PtPd/N-GQDs@Au/GCE (2 h) + PtPd/N-GQDs@Au (~47 h)	Spiked human serum	[49]
GCE	Sandwich immunosensor	Fe_3_O_4_@Ag/GQDs (electrode modifiers) and DAb-AuNPs as labels	CFP-10	DPV (Au^3+^)	0.005–500 mg mL^−1^	0.33 ng mL^−1^	2 h once prepared the CAb/Fe_3_O_4_@Ag/GQD/GCE (~13 h)	Spiked human urine	[57]
SPCE	Sandwich immunosensor	MWCNTs/GQDs (nanocarriers of DAb+HRP)	IL-13sRα2	Amperometry (H_2_O_2_/HQ)	2.7–100 ng mL^−1^	0.8 ng mL^−1^	1 h 45 min + BCAb-Strep/p-ABA/SPCEs (3 h 15 min) + MWCNTs/GQDs-HRP-DAb (~30.5 h)	Raw cellular lysates and extracts of paraffin-embedded tissues from CRC patients	[47]
SPdCE	Sandwich immunosensor	MWCNTs/GQDs (nanocarriers of DAb+HRP)	13sRα2 + CDH-17	Amperometry (H_2_O_2_/HQ)	4.92–100 ng mL^−1^ (IL-13sRα2) 0.11–10 ng mL^−1^ (CDH-17)	1.44 ng mL^−1^ (IL-13sRα2) and 0.03 ng mL^−1^ (CDH-17)	1 h 45 min + BCAb-Strep/p-ABA/SPCEs (3 h 15 min) + MWCNTs/GQDs-HRP-DAb (~30.5 h)	Raw cellular lysates and extracts of paraffin-embedded tissues from CRC patients	[48]
GCE	Sandwich hybridization sensor	ZnFe_2_O_4_/GQDs (Trace label + nanocarrier of Dp)	Target DNA (S2)	DPV (H_2_O_2_/TH)	10^−16^–5 × 10^−9^M	6.2 × 10^−17^ M	160 min + MCH/S1/Pd/GS/GCE (110 min) ZnFe_2_O_4_/GQDs-S3 (11 h)	Spiked human serum	[45]
Au disk electrode (2-mm φ)	Sandwich hybridization sensor	GQDs (platform for HRP immobilization)	miRNA-155	Chronoamperometry (H_2_O_2_/TMB)	1 fM–100 pM	0.14 fM	4 h once prepared the MCH/SHCp/Au (~13 h)	Spiked human serum (1/10 diluted)	[41]
GCE	Direct aptasensing strategy	AgNPs/thiol-GQDs (electrode modifiers)	TNT	DPV (RU)	0.001–0.300 pM	0.33 fM	35 min + AgNPs (2 h) + Thiol-GQDs (54 h) + Ap/AgNPs/thiol-GQD/GCE (>16 h)	Spiked soil and water samples	[59]
Au-SPE	Direct hybridization	CDs (electrode modifiers)	Target DNAs	DPV (safranine)	0.001–20 μM	0.16 nM	1 h + CDs (~3 days) + Cp/CDs/Au-SPEs (~2 days)	DNA isolated from peripheral blood leukocytes from cystic fibrosis patients	[1]
GCE	Direct hybridization	GQDs (electrode modifiers)	Target HBV-DNA	DPV (K_3_[Fe(CN)_6_])	10–500 nM	1 nM	30 min + Cp/GQDs/GCE (12.5 h) + GQDs (50 min)	—	[39]
ITO	Direct hybridization	CM/GQDs (electrode modifiers)	*APOe4* DNA	DPV (CM)	20–400 pg mL^−1^	0.48 pg mL^−1^	— + Cp/CM/GQDs/ITO (>30 min) + GQDs (20 min)	100-fold diluted human blood plasma pre-treated with ammonium sulfate	[58]

p-ABA: p-aminobezoic acid; AXL: receptor tyrosine kinase; CAb: capture antibody; CEA: carcinogenic embryonic antigen; CM: curcumin; Cp: capture probe; CRC: colorectal cancer; CV: cyclic voltammetry; DAb: detector antibody; Dp: detector probe; DPV: differential pulse voltammetry; EIS: electrochemical impedance spectroscopy; GCE: glassy carbon electrode; HCV: hepatitis C virus core antigen; HBV: hepatitis B virus; HQ: hydroquinone; ITO: indium-tin oxide; N-GQDs: nitrogen-doped graphene quantum dots; PAMAM: poly(amidoamine); PtPd/N-GQDs@Au: nanocomposites of N-GQDs, PtPd bimetallic nanoparticles and Au nanoparticles; CFP-10: culture filtrate protein; GS: graphene sheet; MCH: mercaptohexanol; RU: rutin; SPCE: screen-printed carbon electrode; SPdCE: screen-printed dual carbon electrode; Strep: streptavidin; TH: thionine; TMB: 3,3′,5,5′-tetramethylbenzidine; TNT: 2,4,6-Trinitrotoluen.

## References

[1] García-Mendiola T., Bravo I., Lopez-Moreno M., Pariente F., Wannemacher R., Weber K., Popp J., Lorenzo E. (2018). Carbon nanodots based biosensors for gene mutation detection. Sens. Actuator B Chem..

[2] Yáñez-Sedeño P., Campuzano S., Pingarrón J.M. (2017). Carbon nanostructures for tagging in electrochemical biosensing: A Review. Carbon.

[3] Teradal N.L., Jelinek R. (2017). Carbon nanomaterials in biological studies and biomedicine. Adv. Healthc. Mater..

[4] Suvarnaphaet P., Pechprasarn S. (2017). Graphene-Based Materials for Biosensors: A Review. Sensors.

[5] Borenstein A., Hanna O., Attias R., Luski S., Brousse T., Aurbach D. (2017). Carbon-based composite materials for supercapacitor electrodes: A review. J. Mater. Chem. A.

[6] Sun H., Wu L., Wei W., Qu X. (2013). Recent advances in graphene quantum dots for sensing. Mater. Today.

[7] Pedrero M., Campuzano S., Pingarrón J.M. (2017). Electrochemical (bio)sensing of clinical markers using quantum dots. Electroanalysis.

[8] Devi N.R., Vignesh Kumar T.H., Sundramoorthy A.K. (2018). Electrochemically exfoliated carbon quantum dots modified electrodes for detection of dopamine neurotransmitter. J. Electrochem. Soc..

[9] Mehta J., Bhardwaj N., Bhardwaj S.K., Tuteja S.K., Vinayak P., Paul A.K., Kim K.-H., Deep A. (2017). Graphene quantum dot modified screen printed immunosensor for the determination of parathion. Anal. Biochem..

[10] Bhatnagar D., Kaur I., Kumar A. (2017). Ultrasensitive cardiac troponin I antibody based nanohybrid sensor for rapid detection of human heart attack. Int. J. Biol. Macromol..

[11] Chen F., Gao W., Qiu X., Zhang H., Liu L., Liao P., Fu W., Luo Y. (2017). Graphene quantum dots in biomedical applications: Recent advances and future challenges. Front. Lab. Med..

[12] Baluta S., Lesiak A., Cabaj J. (2018). Graphene quantum dots-based electrochemical biosensor for catecholamine neurotransmitters detection. Electroanalysis.

[13] Faridbod F., Sanati A.L. (2019). Graphene quantum dots in electrochemical sensors/biosensors. Curr. Anal. Chem..

[14] Hashemzadeh N., Hasanzadeh M., Shadjou N., Eivazi-Ziaei J., Khoubnasabjafari M., Jouyban A. (2016). Graphene quantum dot modified glassy carbon electrode for the determination of doxorubicin hydrochloride in human plasma. J. Pharm. Anal..

[15] Nguyen H.V., Richter L., Moulick A., Xhaxhiu K., Kudr J., Cernei N., Polansk H., Heger Z., Masarik M., Kopel P., Stiborov M., Eckschlager T., Adam V., Kizek R. (2016). Electrochemical sensing of etoposide using carbon quantum dot modified glassy carbon electrode. Analyst.

[16] Wang L., Tricard S., Yue P., Zhao J., Fang J., Shen W. (2016). Polypyrrole and graphene quantum dots @ Prussian Blue hybrid film on graphite felt electrodes: Application for amperometric determination of L-cysteine. Biosens. Bioelectron..

[17] Jian X., Liu X., Yang H., Guo M., Song X., Dai H., Liang Z. (2016). Graphene quantum dots modified glassy carbon electrode via electrostatic self-assembly strategy and its application. Electrochim. Acta.

[18] Tan F., Cong L., Li X., Zhao Q., Zhao H., Quan X., Chen J. (2016). An electrochemical sensor based on molecularly imprinted polypyrrole/graphene quantum dots composite for detection of bisphenol A in water samples. Sens. Actuators B Chem..

[19] Zhu X., Wu G., Lu N., Yuan X., Li B. (2017). A miniaturized electrochemical toxicity biosensor based on graphene oxide quantum dots/carboxylated carbon nanotubes for assessment of priority pollutants. J. Hazard. Mater..

[20] Lu L., Zhou L., Chen J., Yan F., Liu J., Dong X., Xi F., Chen P. (2018). Nanochannel-confined graphene quantum dots for ultrasensitive electrochemical analysis of complex samples. ACS Nano.

[21] Punrat E., Maksuk C., Chuanuwatanakul S., Wonsawat W., Chailapakul O. (2016). Polyaniline/graphene quantum dot-modified screen-printed carbon electrode for the rapid determination of Cr (VI) using stopped-flow analysis coupled with voltammetric technique. Talanta.

[22] Li Y., Jiang Y., Mo T., Zhou H., Li Y., Li S. (2016). Highly selective dopamine sensor based on graphene quantum dots self-assembled monolayers modified electrode. J. Electroanal. Chem..

[23] Aoun S.B. (2017). Nanostructured carbon electrode modified with N-doped graphene quantum dots–chitosan nanocomposite: A sensitive electrochemical dopamine sensor. R. Soc. Open Sci..

[24] Zhang Y., Wu C., Zhou X., Wu X., Yang Y., Wu H., Guo S., Zhang J. (2013). Graphene quantum dots/gold electrode and its application in living cell H_2_O_2_ detection. Nanoscale.

[25] Ju J., Chen W. (2015). In situ growth of surfactant-free gold nanoparticles on nitrogen-doped graphene quantum dots for electrochemical detection of hydrogen peroxide in biological environments. Anal. Chem..

[26] Xi J., Xie C., Zhang Y., Wang L., Xiao J., Duan X., Ren J., Xiao F., Wang S. (2016). Pd nanoparticles decorated N-doped graphene quantum dots@N-doped carbon hollow nanospheres with high electrochemical sensing performance in cancer detection. ACS Appl. Mater. Interfaces.

[27] Mollarasouli F., Asadpour-Zeynali K., Campuzano S., Yáñez-Sedeño P., Pingarrón J.M. (2017). Non-enzymatic hydrogen peroxide sensor based on graphene quantum dots-chitosan/methylene blue hybrid nanostructures. Electrochim. Acta.

[28] Li Y., Zhang W., Zhang L., Li J., Su Z., Wei G. (2017). Sequence-designed peptide nanofibers bridged conjugation of graphene quantum dots with graphene oxide for high performance electrochemical hydrogen peroxide biosensor. Adv. Mater. Interfaces.

[29] Hu S., Trinchi A., Atkin P., Cole I. (2015). Tunable photoluminescence across the entire visible spectrum from carbon dots excited by white light. Angew. Chem. Int. Ed..

[30] Atkin P., Daeneke T., Wang Y., Careya B.J., Berean K.J., Clark R.M., Ou J.Z., Trinchi A., Cole I.S., Kalantar-zadeh K. (2016). 2D WS_2_/Carbon Dot Hybrids with Enhanced Photocatalytic Activity. J. Mater. Chem. A.

[31] Martínez-Periñán E., Bravo I., Rowley-Neale S.J., Lorenzo E., Banks C.E. (2018). Carbon nanodots as electrocatalysts towards the oxygen reduction reaction. Electroanalysis.

[32] Li H., Chen L., Wu H., He H., Jin Y. (2014). Ionic liquid-functionalized fluorescent carbon nanodots and their applications in electrocatalysis, biosensing, and cell imaging. Langmuir.

[33] Ji H., Zhou F., Gu J., Shu C., Xi K., Jia X. (2016). Nitrogen-doped carbon dots as a new substrate for sensitive glucose determination. Sensors.

[34] Wang Y., Wang Z., Rui Y., Li M. (2015). Horseradish peroxidase immobilization on carbon nanodots/CoFe layered double hydroxides: Direct electrochemistryand hydrogen peroxide sensing. Biosens. Bioelectron..

[35] Huang Q., Hu S., Zhang H., Chen J., He Y., Li F., Weng W., Ni J., Bao X., Lin Y. (2013). Carbon dots and chitosan composite film based biosensor for the sensitive and selective determination of dopamine. Analyst.

[36] Zhang L., Han Y., Zhu J., Zhai Y., Dong S. (2015). Simple and sensitive fluorescent andelectrochemical trinitrotoluene sensors based on aqueous carbon dots. Anal. Chem..

[37] Guo W., Pia F., Zhang H., Sun J., Zhang Y., Sun X. (2017). A novel molecularly imprinted electrochemical sensor modified with carbon dots, chitosan, gold nanoparticles for the determination of patulin. Biosens. Bioelectron..

[38] Zheng W., Wu H., Jiang Y., Xu J., Li X., Zhang W., Qiu F. (2018). A molecularly-imprinted-electrochemical-sensor modified with nanocarbon-dots with high sensitivity and selectivity for rapid determination of glucose. Anal. Biochem..

[39] Xiang Q., Huang J., Huang H., Mao W., Ye Z. (2018). A label-free electrochemical platform for the highly sensitive detection of hepatitis B virus DNA using graphene quantum dots. RSC Adv..

[40] Tuteja S.K., Chen R., Kukkar M., Song C.K., Mutreja R., Singh S., Paul A.K., Lee H., Kim K.-H., Deep A., Suri C.R. (2016). A label-free electrochemical immunosensor for the detection of cardiac marker using graphene quantum dots (GQDs). Biosens. Bioelectron..

[41] Hu T., Zhang L., Wen W., Zhang X., Wang S. (2016). Enzyme catalytic amplification of miRNA-155 detection with graphene quantum dot-based electrochemical biosensor. Biosens. Bioelectron..

[42] Pedrero M., Campuzano S., Pingarrón J.M. (2017). Quantum dots as components of electrochemical sensing platforms for the detection of environmental and food pollutants: A review. J. AOAC Int..

[43] Mollarasouli F., Serafín V., Campuzano S., Yáñez-Sedeño P., Pingarrón J.M., Asadpour-Zeynali K. (2018). Ultrasensitive determination of receptor tyrosine kinase with a label-free electrochemical immunosensor using graphene quantum dotsmodified screen-printed electrodes. Anal. Chim. Acta.

[44] Wei H., Wang E.K. (2013). Nanomaterials with enzyme-like characteristics (nanozymes): Next-generation artificial enzymes. Chem. Soc. Rev..

[45] Liu W., Yang H., Ma C., Ding Y.-N., Ge S., Yu J., Yan M. (2014). Graphene–palladium nanowires based electrochemical sensor using ZnFe_2_O_4_–graphene quantum dots as an effective peroxidase mimic. Anal. Chim. Acta.

[46] Lin L.P., Song X.H., Chen Y.Y., Rong M.C., Zhao T.T., Wang Y.R., Jiang Y.Q., Chen X. (2015). Intrinsic peroxidase-like catalytic activity of nitrogen-doped graphene quantum dots and their application in the colorimetric detection of H_2_O_2_ and glucose. Anal. Chim. Acta.

[47] Serafín V., Valverde A., Martinez-Garcia G., Martinez-Perinan E., Comba F., Garranzo-Asensio M., Barderas R., Yañez-Sedeño P., Campuzano S., Pingarrón J.M. (2019). Graphene quantum dots-functionalized multi-walled carbon nanotubes as nanocarriers in electrochemical immunosensing. Determination of IL-13 receptor α2 in colorectal cells and tumor tissues with different metastatic potential. Sens. Actuators B Chem..

[48] Serafín V., Valverde A., Garranzo-Asensio M., Barderas R., Campuzano S., Yáñez-Sedeño P., Pingarrón J.M. Simultaneous determination of the emerging metastatic biomarkers IL-13Rα2 and CDH-17 using amperometric immunosensors involving grafted screen-printed electrodes and quantum dots/carbon nanotubes as carrier tags for signal amplification. Microchim. Acta.

[49] Yang Y., Liu Q., Liu Y., Cui J., Liu H., Wang P., Li Y., Chen L., Zhao Z., Dong Y. (2017). A novel label-free electrochemical immunosensor based on functionalized nitrogen-doped graphene quantum dots for carcinoembryonic antigen detection. Biosens. Bioelectron..

[50] Dong Y., Shao J., Chen C., Li H., Wang R., Chi Y., Lin X., Chen G. (2012). Blue luminescent graphene quantum dots and graphene oxide prepared by tuning the carbonization degree of citric acid. Carbon.

[51] Gupta S., Smith T., Banaszak A., Boeck J. (2017). Graphene quantum dots electrochemistry and sensitive electrocatalytic glucose sensor development. Nanomaterials.

[52] Muthurasu A., Ganesh V. (2014). Horseradish peroxidase enzyme immobilized graphene quantum dots as electrochemical biosensors. Appl. Biochem. Biotechnol..

[53] Mohammad-Rezaei R., Razmi H. (2016). Preparation and characterization of hemoglobin immobilized on graphene quantum dots-chitosan nanocomposite as a sensitive and stable hydrogen peroxide biosensor. Sens. Lett..

[54] Vasilescu I., Eremia S.A.V., Kusko M., Radoi A., Vasile E., Radu G.L. (2016). Molybdenum disulphide and graphene quantum dots as electrode modifiers for laccase biosensor. Biosens. Bioelectron..

[55] Razmi H., Mohammad-Rezaei R. (2013). Graphene quantum dots as a new substrate for immobilization and direct electrochemistry of glucose oxidase: Application to sensitive glucose determination. Biosens. Bioelectron..

[56] Valipour A., Roushani M. (2017). Using silver nanoparticle and thiol graphene quantum dots nanocomposite as a substratum to load antibody for detection of hepatitis C virus core antigen: Electrochemical oxidation of riboflavin was used as redox probe. Biosens. Bioelectron..

[57] Tufa L.T., Oh S., Tran V.T., Kim J., Jeong K.-J., Park T.J., Kim H.-J., Lee J. (2018). Electrochemical immunosensor using nanotriplex of graphene quantum dots, Fe_3_O_4_, and Ag nanoparticles for tuberculosis. Electrochim. Acta.

[58] Mars A., Hamami M., Bechnak L., Patra D., Raouafi N. (2018). Curcumin-graphene quantum dots for dual mode sensing platform: Electrochemical and fluorescence detection of *APOe4*, responsible of Alzheimer’s disease. Anal. Chim. Acta.

[59] Shahdost-fard F., Roushani M. (2017). Designing an ultra-sensitive aptasensor based on an AgNPs/thiol-GQD nanocomposite for TNT detection at femtomolar levels using the electrochemical oxidation of Rutin as a redox probe. Biosens. Bioelectron..

